# Helping children with reading difficulties: some things we have learned so far

**DOI:** 10.1038/s41539-017-0008-3

**Published:** 2017-03-31

**Authors:** Genevieve McArthur, Anne Castles

**Affiliations:** Department of Cognitive Science, ARC Centre of Excellence in Cognition and its Disorders, Macquarie University, Sydney, NSW 2109 Australia

## Abstract

A substantial proportion of children struggle to learn to read. This not only impairs their academic achievement, but increases their risk of social, emotional, and mental health problems. In order to help these children, reading scientists have worked hard for over a century to better understand the nature of reading difficulties and the people who have them. The aim of this perspective is to outline some of the things that we have learned so far, and to provide a framework for considering the causes of reading difficulties and the most effective ways to treat them.

## Introduction

Over 20 years ago, The Dyslexia Institute asked a 9-year-old boy called Alexander to describe his struggle with learning to read and spell. He bravely wrote: “I have blond her, Blue eys and an infeckshos smill. Pealpie tell mum haw gorgus I am and is ent she looky to have me. But under the surface I live in a tumoyl. Words look like swigles and riting storys is a disaster area because of spellings. There were no ply times at my old school untill work was fineshed wich ment no plytims at all. Thechers sead I was clevor but just didn’t try. Shouting was the only way the techors comuniccatid with me. Uther boys made fun of me and so I beckame lonly and mishroboll”.^1^

Alexander’s experience is not unique. Sixteen per cent of children struggle to learn to read to some extent, and 5% of children have significant, severe, and persistent problems.^2^ The impact of these children’s reading difficulties goes well beyond problems with reading Harry Potter or Snapchat. Poor reading is associated with increased risk for school dropout, attempted suicide, incarceration, anxiety, depression, and low self-concept.^3–6^ It is therefore important to identify and treat poor readers as early as we possibly can.

Scientists have been investigating poor reading—also known as reading difficulty, reading impairment, reading disability, reading disorder, and developmental dyslexia (to name but a few)—for over a century. While it may take another century of research to reach a complete understanding of reading impairment, there are number of things that we have learned about reading difficulties, as well as the children who experience reading them, that provide key clues about how poor reading can be identified and treated effectively.

## Poor readers display different reading behaviours

One thing that we have learned about poor readers is that they are highly heterogeneous; that is, they do not all display the same type of reading impairment (i.e., “reading behaviour”;^7–12^). Some poor readers have a specific problem with learning to read new words accurately by applying the regular mappings between letters and sounds.^7, 8, 13, 14^ This problem, which is often called poor phonological recoding or decoding, can be detected by asking children to read novel “nonwords” such as YIT. Other poor readers have a particular difficulty with learning to read new words accurately that do not follow the regular mappings between letters and sounds, and hence must be read via memory representations of written words.^7, 13, 15, 16^ This problem, which is sometimes called poor sight word reading or poor visual word recognition, can be detected by asking children to read “exception” words such as YACHT. In contrast, some poor readers have accurate phonological recoding and visual word recognition but struggle to read words fluently.^17–19^ Poor reading fluency can be detected by asking children to read word lists or sentences as quickly as they can. In contrast yet again, some poor readers have intact phonological recoding and visual word recognition and reading fluency, but struggle to understand the meaning of what they read. These “poor comprehenders”^20^ can be identified by asking them to read paragraphs aloud (to ascertain that they can read accurately and fluently), and then ask them questions about the meaning of what they have read (to ascertain that they do not understand what they are reading). It is important to note that most poor readers have various combinations of these problems.^21^ For example, Alexander’s spelling suggests that he would have poor phonological decoding (since he misspells words like playtimes as “plytims”) and poor sight word knowledge (since he misspells exception words like said as “sead”). Thus, poor readers vary considerably in the profiles of their reading behaviour.

## Reading behaviours have different “proximal” causes

Another thing we have learned about poor readers is that the same reading behaviour (e.g., inaccurate reading of novel words) does not necessarily have the same “proximal cause”. A proximal cause of a reading behaviour can be defined as a component of the cognitive system that directly and immediately produces that reading behaviour.^22–24^ Most reading behaviours will have more than one proximal cause. Reflecting this, several theoretical and computational models of reading comprise multiple cognitive components that function together to produce successful reading behaviour (e.g., refs 25–28). While these models vary in some respects, all include cognitive components that represent (1) the ability to recognise letters (e.g., S), letter-clusters (e.g., SH), and written words (e.g., SHIP), (2) the ability to recognise and produce speech sounds (e.g., “sh”, “i”, “p”) and spoken words (e.g., “ship”), (3) the ability to access stored knowledge about the meanings of words (e.g., “a floating vessel”), and (4) links between these various components. Impairment in any one of these components or links will directly and immediately impair aspects of reading behaviour. Thus, guided by theoretical and computational models, we have learned that a poor reading behaviour can have multiple proximal causes, and we have some idea about what those proximal causes might be.^10–12^

## Reading behaviours have different “distal” causes

We have also learned that even if two poor readers have exactly the same reading behaviour with exactly the same proximal cause, this reading behaviour will not necessarily have the same “distal cause”. A distal cause has a distant (i.e., an indirect or delayed) impact on a reading behaviour.^22–24^ Distal causes reflect the fact that reading is a taught skill that unfolds over time and across development. It depends upon a range of more cognitive abilities, such as memory, attention, and language skills, to name but a few. Depending on children’s strengths and weaknesses in these underlying abilities, and how these abilities affect learning over time, children will have different profiles of developmental, or distal, causes of their reading impairment. Stated differently, there can be different causal pathways to the same impairment of the reading system.

To provide an example, as mentioned earlier, a common reading behaviour observed in poor readers is inaccurate reading of new or novel words, which can be assessed using nonwords such as YIT. Indeed, some researchers have described this as the defining symptom of reading difficulties.^29^ According to theoretical and computational models of reading, one proximal cause of impaired reading of nonwords is impaired knowledge of letter-sound mappings. But what is responsible for this proximal cause of poor nonword reading? There are multiple hypotheses. The prominent “phonological deficit hypothesis” proposes a pervasive language-based difficulty in processing speech sounds that affects the ability to learn to associate written stimuli (e.g., letters) with speech sounds.^30^ The “paired-associate learning deficit hypothesis” proposes a memory-based difficulty in forming cross-modal mappings across the visual (e.g, letters) and verbal domains (e.g., speech sounds) that affects letter-sound learning (e.g., ref. 31). And the “visual attentional deficit hypothesis” proposes an attention-based impairment in the size of the attentional window, affecting the formation of the sub-word orthographic units (e.g., letters) used in the letter-sound mapping process.^32^ These three hypotheses illustrate why a single reading behaviour (e.g., poor nonword reading) with a common proximal cause (impaired knowledge of letter-sound mappings) might not have the same distal cause (e.g., a phonological deficit, a paired-associate learning deficit, or a visual attention deficit). These hypotheses also raise the possibility that the distal causes of poor readers’ reading behaviours may vary as much (if not more) than the proximal causes and the reading behaviours themselves.

## Poor readers have concurrent problems with their cognition and emotional health

Another thing we have learned about poor readers is that many (but not all) have comorbidities in other aspects of their cognition and emotional health. Regarding cognition, studies have found that a significant proportion of poor readers have impairments in their spoken language.^33–39^ Studies have also found that poor readers have atypically high rates of attention deficit disorder—a neurological problem that causes inattention, poor concentration, and distractibility (e.g., refs 40–42). Regarding emotional health, there is evidence that poor readers, as a group, have higher levels of anxiety than typical readers (e.g., refs 43, 44). The same is true for low self-concept, which can be defined as a negative perception of oneself in a particular domain (e.g., academic self-concept; e.g., refs 45, 46).

The fact that poor readers vary in their comorbid cognitive and emotional health problems—as well as in their reading behaviours, and the proximal and distal impairments of these behaviours—creates an impression of almost overwhelming complexity. However, it is possible to simplify this complexity somewhat using a proximal and distal schema. Specifically, comorbidities of poor reading might be categorised according to whether they represent potential proximal or distal impairment of poor reading—or possibly both. For example, a child’s current problem with spoken vocabulary might be considered a proximal cause of their poor word reading behaviour since, according to theoretical and computational models of reading, vocabulary knowledge may directly underpin word reading accuracy or reading comprehension. However, a child’s previous problem with spoken vocabulary, which may or may not still be present, might be considered a distal cause of their poor word reading: A history of poor understanding of word meanings might reduce a child’s motivation to engage in reading (distal cause), which would impair their development of phonological recoding and visual word recognition (proximal cause), and hence their word reading accuracy and fluency (reading behaviour). Thus, the proximal and distal schema can prove useful in clarifying the causal chain of events linking a reading behaviour to a potential cause.

The proximal and distal schema can also be useful in clarifying reciprocal or circular relationships between comorbidities of poor reading and reading behaviours. For example, if a poor reader has low academic self-concept (distal cause), this may stymie their motivation to pay attention in reading lessons (distal cause), which will impair their learning of letter-sound mappings (proximal cause), and hence their poor word reading (reading behaviour). At the same time, a reverse causal effect may be in play: A child’s poor word reading in the classroom (distal cause) may create a poor perception of their own academic ability (proximal cause) that lowers their academic self-concept (behaviour). Thus, the proximal and distal schema can be used to help develop hypotheses as to whether comorbidities of poor reading are proximal and/or distal causes or consequences of poor reading. Ultimately, of course, all of these hypotheses must be tested through experimental training studies.

## Proximal intervention is more effective than distal intervention

Poor readers have inspired, and have been subjected to, an extraordinary array of interventions such as behavioural optometry, chiropractics, classical music, coloured glasses, computer games, fish oil, phonics, sensorimotor exercises, sound training, spatial frequency gratings, memory training, medication for the inner ear, phonemic awareness, rapid reading, visual word recognition, and vocabulary training, to name just a selection. It is noteworthy that while many of these interventions claim to be “scientifically proven”, few have been tested with a randomised controlled trial (RCT)—an experiment that randomly allocates participants to intervention and control groups in order to reduce bias in outcomes. RCTs are the gold standard method for assessing a treatment of any kind, and the method that must be used to prove the effectiveness of a pharmaceutical treatment.

In order to make sense of the chaotic variety of interventions that claim to help poor readers, it may again be helpful to use the proximal and distal schema outlined above to subdivide interventions into two types: “proximal interventions” that focus training on proximal causes of a reading behaviour that are proposed to be part of the cognitive system for reading (e.g., phonics training, vocabulary training) and “distal interventions” that focus on distal causes of a reading behaviour (e.g., coloured lenses, inner-ear medication). The idea of making a distinction between proximal and distal interventions is supported by the outcomes of a systematic review of all studies that have used an RCT to assess an intervention in poor readers.^47^ These studies assessed the effect of coloured lenses or overlays, medication, motor training, phonemic awareness, phonics, reading comprehension, reading fluency, sound processing, and sunflower therapy on poor readers. One key finding of this review is that it only identified 22 RCTs, which is a small number of gold-standard intervention studies given the huge number of interventions that claim to help poor readers. A second key finding is that the majority of RCTs of interventions for poor readers have assessed the efficacy of phonics training, which trains the ability to use letter-sound mappings to learn to read new or novel words. A third key finding is that only one type of intervention produced a statistically reliable effect. This was phonics training, which focuses on improving a proximal cause of poor word reading (i.e., letter-sound mappings). In contrast, interventions that focused on distal causes of poor reading did not show a statistically reliable effect in poor readers. The outcomes of this systematic review suggest that interventions that focus on phonics—a proximal cause of reading behaviour—are more likely to be effective than interventions that focus on a distal cause. In other words, the “closer” the intervention is to an impaired reading behaviour, the more likely it is to be effective.

## Translating what we know (thus far) into evidence-based practice

At first glance, what we have learned (so far) about poor readers and reading difficulties paints a picture of such complex heterogeneity that it is tempting to throw one’s hands up in despair. And yet, somewhat paradoxically, it is this very heterogeneity that provides some important clues about how to maximise the efficacy of intervention for poor readers. First, the fact that poor readers vary in the nature of their reading behaviours suggests that the first step in identifying an effective intervention for a poor reader is to assess different aspects of reading (e.g., word reading accuracy, reading fluency, and reading comprehension). There are numerous standardized tests provided commercially (e.g., the York Assessment for Reading Comprehension available from GL Assessment)^48^ or for free (e.g., the Castles and Coltheart Word Reading Test—Second Edition (CC2) available at www.motif.org.au)^49^ that can be used to determine if a child falls below the average range for their age or grade for reading accuracy, fluency, or comprehension. In our experience, a teacher who has appropriate training in administrating such tests can carry out this first step effectively.

Second, the fact that poor readers’ reading behaviours can have different proximal causes suggests that the next step is to test them for the potential proximal causes of their poor reading behaviours. This is where cognitive models of reading are a useful roadmap, providing an explicit account of the key processes directly underpinning successful reading behaviour. Again, this can be done using standardized tests that are available commercially (e.g., the Peabody Picture Vocabulary Test Fourth Edition available from Pearson)^50^ or for free (e.g., the Letter-Sound Test available at www.motif.org.au).^51^ And well-trained teachers can administer these tests.

Third, the fact that poor readers vary in the degree to which they experience comorbid cognitive and emotional impairments suggests that it would be useful to assess poor readers for their spoken language abilities, attention, anxiety, depression, and self-concept, at the very least. This knowledge will reveal if they need support in other areas of their development, or if their reading-related intervention needs to be adjusted to accommodate their concomitant impairment in order to maximise efficacy. Trained speech and language therapists typically carry out the assessment of children’s spoken language; neuropsychologists are experts in assessing children’s attention; and clinical psychologists have the expertise to assess children’s emotional health.

Once a poor reader’s reading behaviours, proximal impairments, comorbid cognitive, and emotional health problems have been identified, it should be possible to design an intervention that is a good match to their needs. According to the systematic review conducted by Galuschka et al.^47^, current evidence suggests that this intervention should focus on the proximal impairment of a child’s reading behaviour, rather than a possible distal impairment. Two more recent controlled trials^52, 53^ and a systematic review^54^ further suggest that it is possible to selectively train different proximal impairments of poor reading behaviours in order to improve those behaviours. The outcomes of these studies and reviews tentatively suggest that proximal interventions can be executed by a reading specialist or a highly-sophisticated online reading training programme.

## Summary

In sum, over the last century or so, we have learned important things about reading difficulties and the people who have them. We have learned that poor readers display different reading behaviours, that any one reading behaviour has multiple proximal and distal causes, that some poor readers have concomitant problems in other areas of their cognition and emotional health, and that interventions that focus on proximal causes of poor reading behaviours may be more effective than those that focus on distal causes. This knowledge provides some clues to how we might best assist children with reading difficulties. Specifically, we need to assess poor readers for (1) a range of reading behaviours, (2) proximal causes for each poor reading behaviour, and (3) comorbidities in their cognition and emotional health. It should be possible to design an individualised intervention programme that accommodates for a poor reader’s comorbid cognitive or emotional problems whilst targeting the proximal causes of their poor reading behaviour or behaviours. This approach, which requires the co-ordinated efforts of teachers and specialists and parents, is no mean feat. However, according to the scientific evidence thus far, this is the most effective approach we have for helping children with reading difficulties.

## References

[1] The Dyslexia Institute. *As I See It* (Walker Books, 1990).

[2] Shaywitz SE, Escobar MD, Shaywitz BA, Fletcher JM, Makuch R (1992). Evidence that dyslexia may represent the lower tail of a normal distribution of reading ability. N. Engl. J. Med..

[3] Daniel SS (2006). Suicidality, school dropout, and reading problems among adolescents. J. Learn. Disabil..

[4] McArthur G, Castles A, Kohnen S, Banales E (2016). Low self-concept in poor readers: prevalence, heterogeneity, and risk. Peer. J..

[5] Carroll JM, Maughan B, Goodman R, Meltzer H (2005). Literacy difficulties and psychiatric disorders: evidence for comorbidity. J. Child Psychol. Psychiatry.

[6] Christie CA, Yell ML (2008). Preventing youth incarceration through reading remediation: issues and solutions. Read. Writ. Quart..

[7] Castles A, Coltheart M (1993). Varieties of developmental dyslexia. Cognition.

[8] Goulandris NK, Snowling M (1991). Visual memory deficits: a plausible cause of developmental dyslexia? Evidence from a single case study. Cogn. Neuropsychol..

[9] Jones K, Castles A, Kohnen S (2011). Subtypes of developmental dyslexia: recent developments and directions for treatment. ACQuiring Knowledge Speech Lang. Hear..

[10] McArthur G (2013). Getting to grips with the heterogeneity of developmental dyslexia. Cogn. Neuropsychol..

[11] Peterson R, Pennington B, Olson R (2013). Subtypes of developmental dyslexia: testing predictions of the dual-route and connectionist frameworks. Cognition.

[12] Ziegler JC (2008). Developmental dyslexia and the dual route model of reading: Simulating individual differences and subtypes. Cognition.

[13] Manis FR, Seidenberg MS, Doi LM, McBride-Chang C, Petersen A (1996). On the bases of two subtypes of development dyslexia. Cognition.

[14] Temple CM, Marshall JC (1983). A case study of developmental phonological dyslexia. Br. J. Soc. Psychol..

[15] Friedmann N, Lukov L (2008). Developmental surface dyslexias. Cortex.

[16] Broom YM, Doctor EA (1995). Developmental phonological dyslexia: a case study of the efficacy of a remediation programme. Cogn. Neuropsychol.

[17] de Jong PF, van der Leij A (2003). Developmental changes in the manifestation of a phonological deficit in dyslexic children learning to read a regular orthography. Educ. Psychol.

[18] Landerl K, Wimmer H, Frith U (1997). The impact of orthographic consistency on dyslexia: a German-English comparison. Cognition.

[19] Torgesen, J. K. Recent discoveries on remedial interventions for children with dyslexia. in *The Science of Reading: A Handbook*, (eds Snowling, M. J. & Hulme, C.), pp.521–537 (Blackwell, 2005).

[20] Nation K, Snowling M (1998). Semantic processing and the development of word-recognition skills: evidence from children with reading comprehension difficulties. J. Mem. Lang..

[21] Stuart, M., & Stainthorp, R. *Reading Development and Teaching* (Sage, 2016).

[22] Hulme, C., & Snowling, M. *Developmental Disorders of Language, Learning and Cognition* (Wiley-Blackwell, 2009).

[23] Jackson, N. E., & Coltheart, M. *Routes to Reading Success and Failure: Toward an Integrated Cognitive Psychology of Atypical Reading* (Psychology Press, 2001).

[24] Castles A, Kohnen S, Nickels L, Brock J (2014). Developmental disorders: what can be learned from cognitive neuropsychology?. Phil. Trans. R. Soc. B.

[25] Coltheart M, Rastle K, Perry C, Langdon R, Ziegler J (2001). DRC: a dual route cascaded model of visual word recognition and reading aloud. Psychol. Rev..

[26] Hoover WA, Gough PB (1990). The simple view of reading. Read. Writ..

[27] Perry C, Ziegler JC, Zorzi M (2007). Nested incremental modeling in the development of computational theories: the CDP+ model of reading aloud. Psychol. Rev..

[28] Plaut DC, McClelland JL, Seidenberg MS, Patterson K (1996). Understanding normal and impaired word reading: computational principles in quasi-regular domains. Psychol. Rev..

[29] Rack JP, Snowling MJ, Olson RK (1992). The nonword reading deficit in developmental dyslexia: a review. Read. Res. Q..

[30] Snowling M (1998). Dyslexia as a phonological deficit: evidence and implications. Child Psychol. Psychiatry.Rev..

[31] Warmington M, Hulme C (2012). Phoneme awareness, visual-verbal paired-associate learning, and rapid automatized naming as predictors of individual differences in reading ability. Sci. Studying Read..

[32] Bosse M-L, Tainturier M, Valdois S (2007). Developmental dyslexia: the visual attention span deficit hypothesis. Cognitio.

[33] Bishop DVM, Snowling MJ (2004). Developmental dyslexia and specific language impairment: same or different?. Psychol. Bull..

[34] Eisenmajer N, Ross N, Pratt C (2005). Specificity and characteristics of learning disabilities. J. Child Psychol. Psychiatry..

[35] Fraser J, Goswami U, Conti-Ramsden G (2010). Dyslexia and specific language impairment: the role of phonology and auditory processing. Sci. Studies Read..

[36] McArthur G, Castles A (2013). Phonological processing deficits in specific reading disability and specific language impairment: same or different?. J. Res. Read..

[37] McArthur GM, Hogben JH, Edwards VT, Heath SM, Mengler ED (2000). On the “specifics” of specific reading disability and specific language impairment. J Child Psychol. Psychiatry Allied Disciplines.

[38] Rispens J, Been P (2007). Subject–verb agreement and phonological processing in developmental dyslexia and specific language impairment (SLI): a closer look. Int.J. Lang. Commun.Dis..

[39] Catts HW, Adolf SM, Hogan TP, Weismer SE (2005). Are specific language impairment and dyslexia distinct disorders?. J. Speech. Lang. Hear. Res..

[40] Gilger JW, Pennington BF, DeFries JC (1992). A twin study of the etiology of comorbidity: attention-deficit hyperactivity disorder and dyslexia. J. Am. Acad. Child Adolesc. Psychiatry.

[41] Shaywitz BA, Fletcher JM, Shaywitz SE (1995). Defining and classifying learning disabilities and attention-deficit/hyperactivity disorder. J. Child. Neurol..

[42] Willcutt EG, Pennington BF (2000). Comorbidity of reading disability and attention-deficit/hyperactivity disorder differences by gender and subtype. J. Learn. Disabil..

[43] Maughan B, Carroll J (2006). Literacy and mental disorders. Curr. Opin. Psychiatry.

[44] Mugnaini D, Lassi S, La Malfa G, Albertini G (2009). Internalizing correlates of dyslexia. World J. Clin. Pediatr..

[45] Snowling MJ, Muter V, Carroll J (2007). Children at family risk of dyslexia: a follow-up in early adolescence. J. Child Psychol. Psychiatry.

[46] Taylor LM, Hume IR, Welsh N (2010). Labelling and self‐esteem: the impact of using specific vs. generic labels. Educ. Psychol..

[47] Galuschka K, Ise E, Krick K, Schulte-Körne G (2014). Effectiveness of treatment approaches for children and adolescents with reading disabilities: a meta-analysis of randomized controlled trials. PLoS ONE.

[48] Snowling, M. J. et al. *YARC York Assessment of Reading for Comprehension Passage Reading* (GL Assessment, 2009).

[49] Castles A (2009). Assessing the basic components of reading: a revision of the Castles and Coltheart test with new norms. Aust. J. Learn. Diffic..

[50] Dunn, L. M., & Dunn, D. M. *Peabody Picture Vocabulary Test: PPVT 4* (Pearson, 2015).

[51] Larsen L, Kohnen S, Nickels L, McArthur G (2015). The letter-sound test (LeST): a reliable and valid comprehensive measure of grapheme-phoneme knowledge. Aust. J. Learn. Diffic..

[52] McArthur G (2015). Sight word and phonics training in children with dyslexia. J. Learn. Disabil..

[53] McArthur G (2015). Replicability of sight word training and phonics training in poor readers: a randomised controlled trial. Peer. J..

[54] McArthur, G. et al. Phonics training for English‐speaking poor readers. *The Cochrane Library*. **12**, 1–102 (2012). 10.1002/14651858.CD009115.pub2 23235670

